# A Revised LRSPR Sensor with Sharp Reflection Spectrum

**DOI:** 10.3390/s140916664

**Published:** 2014-09-05

**Authors:** Yinquan Yuan, Yutang Dai

**Affiliations:** National Engineering Laboratory for Fiber Optic Sensing Technology, Wuhan University of Technology, Wuhan 430070, China; E-Mail: daiyt6688@whut.edu.cn

**Keywords:** optical sensor, surface plasmon resonance, waveguide-coupled, multi-layer model

## Abstract

In this work, we have proposed a novel long-range surface plasmon resonance (LRSPR) sensor with sharp reflection spectrum, which consists of a glass prism, a (A/B)^4^-type waveguide-coupled layer and a metal layer. To reveal its sharp reflection spectrum perfectly, we have simulated the effects of all factors of this LRSPR sensor on the reflection spectrum, and finally presented the optimal parameters of the LRSPR sensor with sharp reflection spectrum.

## 1. Introduction

Surface plasmon resonance (SPR) refers to the optical excitation of surface plasmon wave at the interface between a noble metal and a dielectric. The SPR sensors have great potential in chemical and biological sensing, including the measurements on the concentration of an analyte in a complex sample, the specificity, affinity and biomolecular interaction kinetics [1–3]. The most common SPR sensor, also called the conventional SPR (CSPR) sensor, is in Kretschmann configuration and comprises of a glass prism, a thin metal film and a sample in sequence, that may work in angular or wavelength interrogation mode. For the SPR sensor in angular interrogation mode, the wavelength of the entrance light is constant and the incident angle can be adjusted [4–6]. Until now, numerous works have been done to develop the CSPR sensors in angular interrogation mode [7–11], but these CSPR sensors have a common disadvantage, namely the wide reflection spectrum (∼2–3° of dip width) that produces low sign-to-noise ratio.

Apart from CSPR sensors, other three kinds of SPR sensors have been developed also [12]. The first kind is waveguide-coupled SPR (WCSPR) sensor, whose configuration is made up of a prism, a metal layer, a dielectric waveguiding layer, a metal layer and a sample in sequence [13–15]. In the reflection spectrum of WCSPR sensor, there are one CSPR dip, one WCSPR dip and several waveguide-coupled resonance dips. Although the WCSPR dip is one sharp dip (∼0.3°), it is difficult to distinguish those dips in the measurements. The second kind is plasmon-waveguide SPR (PWSPR) sensor, whose configuration is made up of a prism, a metal layer, a dielectric waveguiding layer and a sample in sequence [16,17]. In its reflection spectrum, there are one CSPR dip and one sharp WCSPR dip (∼0.5°), but our simulations have shown that the refractive index (RI) range of the WCSPR dip is too small.

The third kind is a long-range SPR (LRSPR) sensor, whose configuration is made up of a prism, a dielectric layer, a metal layer and a sample in sequence [18–21]. Based on LRSPR structure, we have proposed a revised LRSPR sensor with sharp reflection spectrum, which consists of a glass prism, a (A/B)^4^-type waveguide-coupled (WC) layer and a metal layer. To reveal its sharp reflection spectrum perfectly, we have investigated the effects of key parameters of the revised LRSPR sensor on the reflection spectrum, and finally presented the optimal parameters.

## 2. Model and Simulation Results

**Figure 1.**
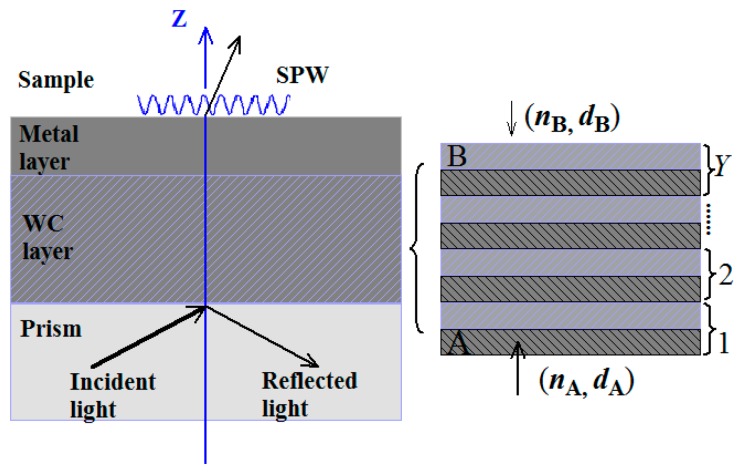
Configuration of the revised long-range surface plasmon resonance (LRSPR) sensor.

Figure 1a gives the configuration of the revised LRSPR sensor. The first layer is the coupling prism with the RI of *n*_p_ = 1.5105. As shown in Figure 1b, the next is the WC layer with the periodic structure of type (A/B)*^Y^*, where A = TiO_2_ and B = SiO_2_, *Y* denotes the number of A/B layer, the RI and thickness of A layer is denoted by *n*_2_ (=2.232) and *d*_2_, the RI and thickness of B layer denoted by *n*_3_ (=1.451) and *d*_3_ respectively. Let *N* = 2*Y* + 3, then the (*N*−1)th layer is the noble metal layer whose dielectric function obeys Lorentz-Drude model and can be expressed in the following form [22]

(1) $${ɛ}_{\text{m}}(\omega )=1-\frac{f_{0}\omega_{\text{p}}^{2}}{\omega (\omega +i\Gamma_{0})}+\sum_{j=1}^{k}\frac{f_{j}\omega_{\text{p}}^{2}}{\omega_{j}^{2}-\omega^{2}+i\omega \Gamma_{0}}$$

where *ω* = 2πc/λ is the light frequency, *ω*_p_ the plasma frequency, *k* the number of oscillators with frequency *ω_j_*, strength *f_j_*, and lifetime 1/*Γ_j_*, while *f*_0_*ω*_p_^2^ is the plasma frequency associated with intraband transitions with oscillator strength *f*_0_ and damping constant *Γ*_0_. The *N*th layer is the sample with the RI denoted by *n*_sam_.

**Figure 2.**
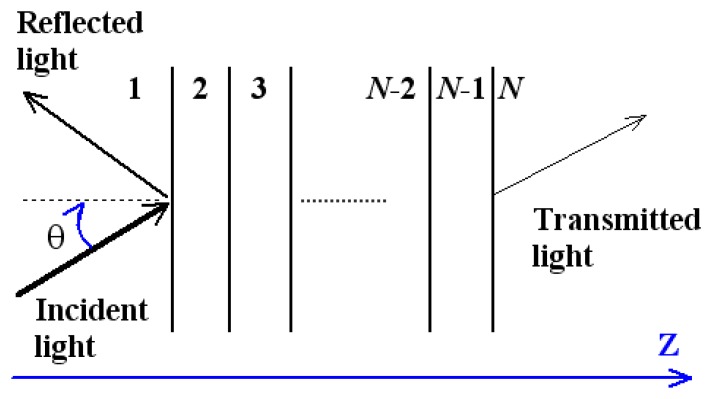
Multi-layer model of the surface plasmon resonance (SPR) sensor.

To calculate the reflected light intensity of *p*-polarized incident light, the *N*-layer model has been used to simulate the SPR sensor working in the angle interrogation mode. As shown in Figure 2, these layers are stacked along the *z*-axis, any layer is defined by the thickness *d*_k_, dielectric constant *ɛ*_k_, and refractive index *n*_k_, where *k* is a footnote that denotes the prism, metal, dielectric layers and the sample. All layers are assumed to be uniform, isotropic and non-magnetic. The tangential fields at the first boundary *Z* = *Z*_1_ = 0 are related to those at the final boundary *Z* = *Z_N_*_−1_ by [3,22]

(2) $$\left[\begin{array}{c} U_{1}\\ V_{1} \end{array}\right]=M\left[\begin{array}{c} U_{N-1}\\ V_{N-1} \end{array}\right]$$

where *U*_1_ and *V*_1_, respectively, are the tangential components of electric and magnetic fields at the boundary of first layer, *U_N_*_−1_ and *V_N_*_−1_ are the corresponding fields at the boundary of *N*th layer, *M* is known as characteristic matrix of the combined structure and is given by

(3) $$M=\prod_{k=2}^{N-1}M_{k}$$

with

(4) $$M_{k}=\left[\begin{array}{cc} \cos\beta_{k} & -\text{i}\;\sin\beta_{k}/q_{k}\\ -\text{i}q_{k}\sin\beta_{k} & \cos\beta_{k} \end{array}\right]$$

where

(5) $$q_{k}=\frac{{\left({ɛ}_{k}-n_{1}^{2}\sin^{2}\theta_{1}\right)}^{1/2}}{{ɛ}_{k}}$$

(6) $$\beta_{k}=\frac{2\pi d_{k}}{\lambda }{\left({ɛ}_{k}-n_{1}^{2}\sin^{2}\theta_{1}\right)}^{1/2}$$

Here *λ* is the free space wavelength. The amplitude reflection coefficient for *p*-polarized incident wave is given by

(7) $$r_{\text{p}}=\frac{\left(M_{11}+M_{12}q_{N})q_{1}-(M_{21}+M_{22}q_{N}\right)}{\left(M_{11}+M_{12}q_{N})q_{1}+(M_{21}+M_{22}q_{N}\right)}$$

Finally, the reflection intensity for *p*-polarized light is

(8) $$R_{\text{p}}=|r_{\text{p}}{|}^{2}$$

**Figure 3.**
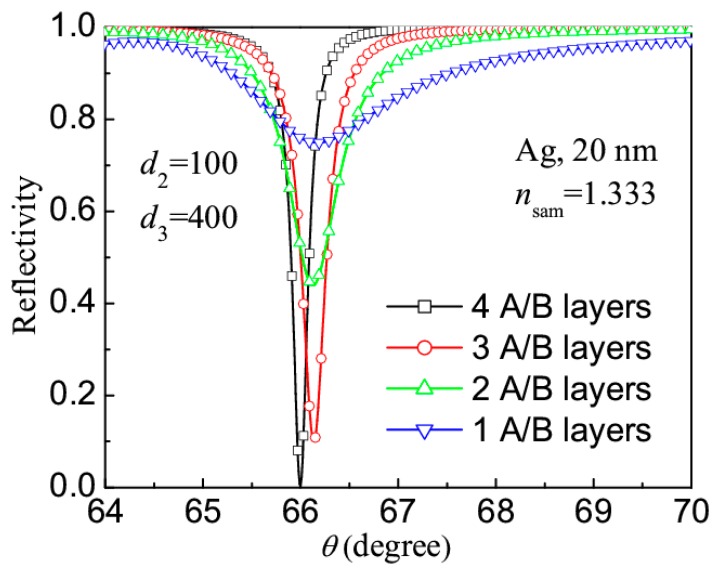
Reflection spectra of the revised LRSPR sensors with different numbers of A/B layers.

Firstly, to understand the effect of the number of A/B layers on the SPR, Figure 3 depicts the reflection spectra of the revised LRSPR sensors with different number of A/B layers (1, 2, 3 and 4), where the RI *n*_sam_ = 1.3330, the thicknesses *d*_2_ = 100 nm and *d*_3_ = 400 nm were chosen. It can be seen that, the LRSPR sensor with one A/B layer has flat resonance spectrum (full width at half minimum, FWHM Δ*θ*_FWHM_ = 1.81°), the sensor with three A/B layers has a better resonance dip (Δ*θ*_FWHM_ = 0.30°) and the sensor with four A/B layers has a sharpest resonance dip (Δ*θ*_FWHM_ = 0.19°). We have also calculated the reflection spectra of the LRSPR sensors with five and more A/B layers, and found that their reflection spectra have no obvious improvement. In addition, the increase of A/B layers causes the preparation difficulty of the LRSPR sensor, so we take *Y* = 4 in the following discussion.

**Figure 4.**
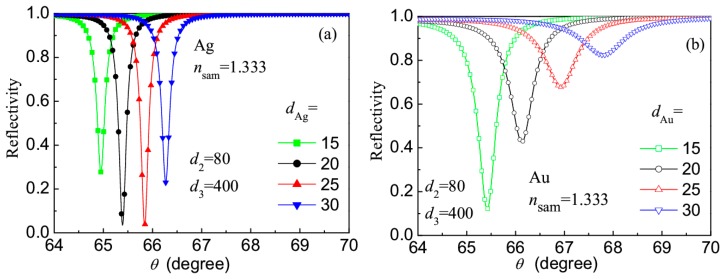
Reflection spectra of the revised LRSPR sensors with Ag or Au layer.

Then we have investigated the effects of different metals and thicknesses for the LRSPR sensor with 4 A/B layers. Figure 4 gives the reflection spectra of the sensors corresponding to metals Ag and Au with different thicknesses, where *n*_sam_ = 1.333, *d*_2_ = 80 nm and *d*_3_ = 400 nm were chosen. For the sensors with Ag layer (Figure 4a), when the thickness of Ag layer changes from 15 nm to 30 nm, the resonance angle (*θ*_res_) increases in the range of 64.9–66.3°, all the resonance dips are very sharp, and the best choice is the Ag layer of 20 nm. When using Au to prepare the metal layer of the sensor (Figure 4b), the resonance dips are wider, the signal-to-noise ratio (Δ*θ*_res_/Δ*θ*_FWHM_) is lower, and it is recommended that its thickness should not be greater than 20 nm.

**Figure 5.**
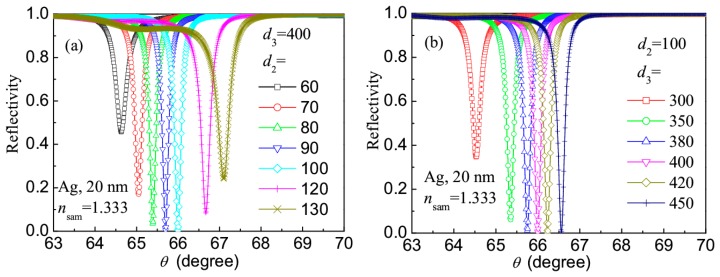
Effects of thicknesses *d*_2_ and *d*_3_ on the reflection spectrum of the revised LRSPR sensor.

Coming from above discussions, better reflection spectra can be obtained by using the Ag layer of 20 nm, so such a metal layer was fixed in the following. Figure 5 shows the effects of the thicknesses *d*_2_ and *d*_3_ on the reflection spectrum of the revised LRSPR sensor, where *n*_sam_ = 1.333 nm, *d*_3_ = 400 nm (a) or *d*_2_ = 100 nm (b) were fixed. It can be seen that, when *d*_2_ is in the range of 70–120 nm and *d*_3_ in the range of 300–450 nm, the reflection spectrum has better resonance dip.

**Figure 6.**
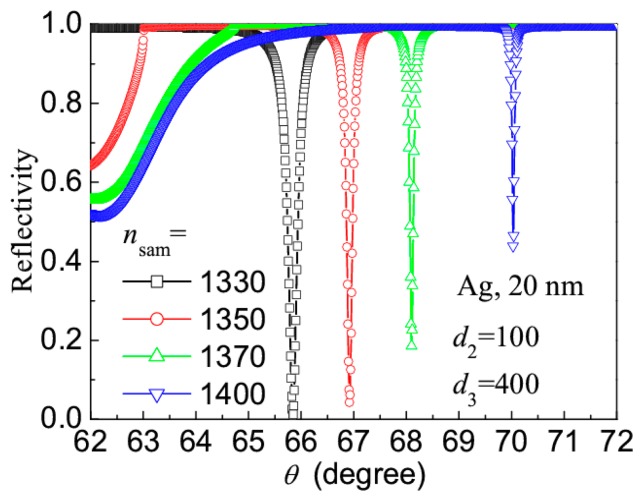
Reflection spectra of the revised LRSPR sensor corresponding to different sample RIs.

Finally, to understand the sensitivity (Δ*θ*_res_/Δ*n*_sam_) and signal-to-noise ratio (Δ*θ*_res_/Δ*θ*_FWHM_) of the revised LRSPR sensor, Figure 6 shows the effect of sample RI on the reflection spectrum of the revised LRSPR sensor, where *d*_2_ = 100 nm and *d*_3_ = 400 nm were fixed. When the sample RI increases from 1.330 to 1.400, the resonance angle increases linearly from 65.85° to 70.05°, the sensitivity Δ*θ*_res_/Δ*n*_sam_ is 60°/RIU approximately. Moreover, their spectrum width (Δ*θ*_FWHM_ < 0.2°) is smaller than one 10th of the spectrum width (Δ*θ*_FWHM_ > 2°) of the CSPR sensor, so their signal-to-noise ratio (Δ*θ*_res_/Δ*θ*_FWHM_) is 10 times greater than that of the CSPR sensor.

## 3. Conclusions

We have proposed a revised LRSPR sensor with sharp reflection spectrum, it consists of a glass prism, a (A/B)^4^-type waveguide-coupled layer and a silver layer. Using the Ag layer with the thickness of about 20 nm, when the thickness of A layer (TiO_2_, RI = 2.232) is in the range of 70–120 nm and the thickness of B layer (SiO_2_, RI = 1.451) is in the range of 300–450 nm, the reflection spectrum of the revised LRSPR sensor has a better resonance dip with the FWHM of about 0.2°, which is smaller than one 10th of the spectrum width of the CSPR sensor. Moreover, when the sample RI increases from 1.330 to 1.400, the resonance angle increases linearly from 65.85° to 70.05° and the sensitivity of 60°/RIU was obtained.
